# Pathologic Response Rates of Gemcitabine/Cisplatin versus Methotrexate/Vinblastine/Adriamycin/Cisplatin Neoadjuvant Chemotherapy for Muscle Invasive Urothelial Bladder Cancer

**DOI:** 10.1155/2013/317190

**Published:** 2013-12-08

**Authors:** Franklin C. Lee, William Harris, Heather H. Cheng, Jaideep Shenoi, Song Zhao, Junfeng Wang, Thomas Champion, Jason Izard, John L. Gore, Michael Porter, Evan Y. Yu, Jonathan L. Wright

**Affiliations:** ^1^Department of Urology, University of Washington School of Medicine, Seattle, WA 98195, USA; ^2^Department of Urology, University of Washington Medical Center, Health Sciences Building, 1959 NE Pacific, BB-1115, Box 356510, Seattle, WA 98195, USA; ^3^Division of Medical Oncology, Department of Medicine, University of Washington School of Medicine, Seattle, WA 98195, USA; ^4^Clinical Research Division, Fred Hutchinson Cancer Research Center, Seattle, WA 98109, USA; ^5^Group Health Permanente, Seattle, WA 98109, USA; ^6^Huntsman Cancer Institute, Division of Medical Oncology, The University of Utah, Salt Lake City, UT 84112, USA; ^7^Public Health Sciences, Fred Hutchinson Cancer Research Center, Seattle, WA 98109, USA

## Abstract

*Objectives*. To compare pathologic outcomes after treatment with gemcitabine and cisplatin (GC) versus methotrexate, vinblastine, adriamycin, and cisplatin (MVAC) in the neoadjuvant setting. *Methods*. Data was retrospectively collected on 178 patients with T2-T4 bladder cancer who underwent radical cystectomy between 2003 and 2011. Outcomes of interest included those with complete response (pT0) and any response (≤pT1). Odds ratios were calculated using multivariate logistic regression. *Results*. Compared to those who did not receive neoadjuvant chemotherapy, there were more patients with complete response (28% versus 9%, OR 3.11 (95% CI: 1.45–6.64), *P* = 0.03) and any response (52% versus 25%, OR 3.23 (95% CI: 1.21–8.64), *P* = 0.01). Seventy-two patients received GC (*n* = 41) or MVAC (*n* = 31). CR was achieved in 29% and 22% of GC and MVAC patients, respectively (multivariate OR 0.39, 95% CI 0.10–1.58). Any response (≤pT1) was achieved in 56% of GC and 45% of MVAC patients (multivariate OR 0.45, 95% CI 0.12–1.71). *Conclusions*. We observed similar pathologic response rates for GC and MVAC neoadjuvant chemotherapy in this cohort of patients with muscle invasive urothelial cancer (MIBC). Our findings support the use of GC as an alternative regimen in the neoadjuvant setting.

## 1. Introduction

Bladder cancer, the fourth most common cancer in men, is commonly treated with radical cystectomy in patients with muscle invasive urothelial bladder cancer (MIBC). Survival is strongly correlated with both tumor and nodal stage with five-year survival of 15–35% in those with lymph node positive disease [1]. In patients treated with radical cystectomy, pathologic stage classification is higher than predicted clinical stage classification in 42% of patients [1]. Furthermore, occult lymph node disease is identified in 20–45% of patients at the time of cystectomy with clinical T2-T4 disease [1–3]. In addition, distant recurrences are seen in 20–50% of patients and outnumber local recurrences, which are seen in 5–15% of patients [2, 3]. Neoadjuvant chemotherapy is thought to provide additional benefit by treatment of “micrometastases” present in large numbers of patients with clinically localized disease.

Several studies have evaluated the role of platinum-based neoadjuvant chemotherapy prior to radical cystectomy. Level 1 evidence supports use of neoadjuvant chemotherapy with a demonstrated 33% reduction in the risk of death among patients that received combination therapy compared with those that received surgery alone [4]. A meta-analysis further confirmed the benefit of platinum-based neoadjuvant chemotherapy with a 14% relative reduction in risk of disease-specific mortality (HR 0.86, 95% CI 0.77–0.95) over cystectomy [5]. The greatest benefit is observed in patients who, at time of cystectomy, are found to have achieved a pathologic complete response (pT0) which has since been established as an intermediate surrogate for survival [4, 6, 7]. For these reasons, neoadjuvant chemotherapy is an important consideration in the management of patients with MIBC undergoing radical cystectomy [8].

While many studies have focused on pathologic complete responders and the survival benefit derived from complete response, those who have any pathologic response also appear to benefit from neoadjuvant chemotherapy [9, 10]. The importance of downstaging resulting from neoadjuvant chemotherapy was demonstrated in a study comparing nonresponders (pT2 or greater at RC) with responders (pT1 or less at RC) [10]. Two years after RC, the responders had a significantly higher disease-free rate (91% versus 37%) [10]. Similarly, in a retrospective review of Nordic Cystectomy Trials 1 and 2, patients with residual noninvasive cancer at time of cystectomy (pTa and pTis) who received neoadjuvant chemotherapy had significantly improved disease-specific survival compared with those with noninvasive disease at cystectomy who had not received neoadjuvant chemotherapy [9].

Data has consistently demonstrated the benefit of cisplatin-based chemotherapy for MIBC in the neoadjuvant setting, but there is little data regarding the optimal cisplatin-based regimen [4, 5, 7]. Methotrexate, vinblastine, doxorubicin, and cisplatin (MVAC) based neoadjuvant chemotherapy is the only regimen with level 1 data [4]. Phase III data comparing gemcitabine/cisplatin (GC) and MVAC chemotherapy in patients with locally advanced or metastatic bladder cancer (Stage T4bN2N3) demonstrated no significant outcome differences between the two regimens [11, 12]. Overall survival (HR: 1.04; 95% CI 0.82–1.32 *P* = 0.75) and response rates (GC: 54% MVAC: 55%) were similar between GC and MVAC [12]. GC was better tolerated than MVAC with 63% of patients receiving GC without dose adjustments compared to only 37% of those on MVAC therapy. Patients on GC were also found to have significantly less grade 3-4 anemia (GC: 57%; MVAC: 21%), neutropenic fever (GC: 2%; MVAC: 14%), neutropenic sepsis (GC: 1%; MVAC: 12%), and grade 3-4 mucositis (GC: 1%; MVAC: 22%). Efficacy between GC and MVAC was similar (median survival of 15.4 months and 16.1 months, resp.); however neutropenia, neutropenic fever, mucositis, and anemia in those receiving GC were significantly decreased [12].

As a result of this data, many have adopted GC in the neoadjuvant setting. In this study, we retrospectively compare GC and MVAC response rates in patients with MIBC undergoing cystectomy.

## 2. Methods

### 2.1. Study Population

After obtaining institutional review board approval, we retrospectively identified patients treated with radical cystectomy at our institution beginning in September 2003. This date was chosen to correspond with the publication of the Southwest Oncology Group-8710 neoadjuvant chemotherapy study of MVAC in patients with MIBC. Patients were included if they had clinical organ confined T2-T4N0 urothelial bladder cancer and were treated with neoadjuvant chemotherapy. Those with variant histology in the primary tumor were included only if the predominant histology was urothelial carcinoma. Those with micropapillary or small cell/neuroendocrine tumors were excluded. Patients undergoing salvage cystectomy following chemotherapy and radiation were also excluded.

### 2.2. Data Collection and Coding

Demographic and disease characteristics were collected from the medical records. Demographic data included age at treatment, gender, and race (Caucasian versus other). Clinical data included comorbid conditions (e.g., diabetes mellitus, cardiac disease, liver disease, and pulmonary disease) and baseline creatinine. Disease characteristics included clinical stage, prior BCG therapy, and extent of endoscopic tumor debulking by transurethral resection of bladder tumor (TURBT) prior to chemotherapy. Clinical T-stage classification was derived from the surgical pathology from the TURBT and cross-sectional imaging. Pathologic T-stage classification was derived from surgical specimens following neoadjuvant chemotherapy and radical cystectomy. For patients receiving neoadjuvant chemotherapy, the type of regimen was recorded as GC, MVAC, and others. The primary outcome of interest was pathologic complete response (pT0); we secondarily evaluated pathologic downstaging to ≤pT1.

### 2.3. Statistical Analysis

We first compared differences between patients grouped by whether or not they received neoadjuvant chemotherapy. Chi-squared tests were used to compare demographic, clinical, and pathologic differences. Multivariate logistic regression analysis (adjusting for age, gender, race, clinical stage, prior BCG use, extent of TURBT, cardiac disease, and baseline creatinine) was then performed to determine the odds ratio and 95% CI for complete (pT0) or any pathologic response (≤pT1) based on receiving neoadjuvant chemotherapy. We then limited analysis to those receiving GC or MVAC neoadjuvant chemotherapy. Owing to small numbers, we did not include other regimens in this analysis. Chi-squared tests and multivariate logistic analysis were performed as above comparing the GC and MVAC groups. All analyses were done with Stata/SE 12 software (College Station, TX).

## 3. Results

Of the 178 patients identified with clinically localized MIBC, 87 (49%) received neoadjuvant chemotherapy. Compared with those who did not receive neoadjuvant chemotherapy, patients treated with neoadjuvant chemotherapy were younger (age < 70), but otherwise the demographic and clinical characteristics were similar (Table 1). Complete pathologic response was more common among those receiving neoadjuvant chemotherapy (Table 2; 28% versus 9%, respectively; OR 3.11 [95% CI: 1.45–6.64]; *P* = 0.03), as was any pathologic response (52% versus 25%, respectively; OR 3.23 [95% CI: 1.21–8.64]; *P* = 0.01).

We identified 72 patients who received either GC (57%) or MVAC (43%) in the neoadjuvant setting. Fifteen patients (8.4%) received other chemotherapeutic regimens (Table 3). The vast majority of patients over the age of 70 received GC (14/15). The baseline creatinine was ≤1.0 in 68% and 53% of GC and MVAC patients, respectively (*P* = 0.24). In Table 3, clinical and pathologic data are shown. We found no difference in clinical stage (*P* = 0.52) or in the distribution of pathologic stage (*P* = 0.45).

Pathologic complete responses (pT0) were achieved in 29% and 22% of GC and MVAC patients, respectively (Table 4). Any pathologic response (≤pT1) was seen in 49% and 35% of GC and MVAC patients, respectively. In multivariate analysis adjusting for age, gender, race, clinical T and N stage, completion of diagnostic TURBT, and baseline creatinine, there was no significant difference in complete (OR 0.39, 95% CI 0.10–1.58; *P* = 0.52) or any pathologic response (OR 0.45, 95% CI 0.12–1.71; *P* = 0.26) between those who received GC or MVAC.

## 4. Discussion

Use of cisplatin-based neoadjuvant chemotherapy prior to radical cystectomy for treatment of clinically localized MIBC is supported by level 1 evidence [4, 5, 7]. Current utilization of neoadjuvant chemotherapy is low, with only 9% of eligible patients in North American academic centers receiving neoadjuvant cisplatin-based chemotherapy [13]. Our study demonstrates that a center aggressive in the use of neoadjuvant chemotherapy with 49% of eligible patients receiving neoadjuvant chemotherapy can yield high rates of complete response (28%) and any response (52%). Furthermore, we found no difference in pathologic response rates (complete or any) between the two most common neoadjuvant chemotherapy regimens, GC and MVAC.

The International Collaboration of Trialists randomized 976 patients with cT2-T4N0 muscle invasive urothelial bladder cancer to cisplatin, methotrexate, or vinblastine prior to local therapy. While local therapy was heterogeneous and consisted of radiotherapy, radical cystectomy, or a combination of both, there was a 10-year overall survival benefit in favor of cisplatin based neoadjuvant chemotherapy (36% versus 30%; HR 0.84; *P* = 0.037) [12]. Those that underwent radical cystectomy after neoadjuvant chemotherapy were found to have a 26% reduction in risk of death [14].

The Southwest Oncology Group (SWOG) 8710 trial randomized 317 patients to cystectomy alone or MVAC plus cystectomy [4]. This study demonstrated a 33% (HR 1.33; 95% CI 1.0–1.76) greater risk of death in patients treated with cystectomy alone with an improvement in median survival from 46 months (with cystectomy alone) to 77 months (with MVAC + cystectomy) [4]. Based on these trials demonstrating efficacy in cisplatin based chemotherapy, the primary regimens used are GC and MVAC with debate over the comparative efficacy. In this retrospective study, we found no difference in the response rates between GC and MVAC.

One of the major concerns regarding the use of neoadjuvant MVAC therapy is toxicity, which includes neutropenia (10% neutropenic fever, 6% sepsis), mucositis (17% grades 3 and 4), and renal, cardiac, and neurologic toxicities. It is also associated with a toxic death rate of 3-4% [11, 12]. Neoadjuvant regimens such as GC in the locally advanced/metastatic setting have reported similar efficacy to MVAC with lower toxicity [11, 12]. In phase III clinical trials, patients that received GC had lower toxicity related mortality (3% versus 1%) with significantly lower rates of neutropenic fevers (14% versus 2%) and mucositis (22% versus 1%) compared to MVAC [11, 12]. For this reason, GC is frequently used as an alternative to MVAC therapy. Furthermore, patients on GC had improved weight, performance status, and fatigue during treatment [11, 12], important factors given that patients with bladder cancer are often older with multiple medical comorbidities.

Several, but not all, retrospective analyses have demonstrated similar efficacy between GC and MVAC regimens with varying proportions of those having a response [15–18]. Weight et al. retrospectively reviewed 117 patients, 29 (25%) of which received neoadjuvant chemotherapy [19]. The majority of the patients that received neoadjuvant chemotherapy received GC (20 patients; 69%) and only 5 patients (17%) received MVAC [19]. In their analysis only 2 patients achieved pathologic complete response [19]. Based on their findings, Weight and colleagues concluded that GC may be an inferior regimen to traditional MVAC chemotherapy. This is in stark contrast to the data found in this paper and others. Yeshchina and colleagues retrospectively reviewed 61 patients who were treated with neoadjuvant chemotherapy. Sixteen patients received GC and 45 received MVAC with a decreased but nonsignificant difference in pT0 rates in GC (25%) and MVAC (31%) [15]. In another study, Fairey et al. retrospectively analyzed 116 patients, 58 of which received GC neoadjuvant chemotherapy and 58 received MVAC neoadjuvant chemotherapy. They found a greater but nonstatistically significant difference in pathologic complete response rates for GC (27%) compared to MVAC (17%) [16]. Dash et al. found nearly identical pT0 rates for patients who received GC (42) and MVAC (54) neoadjuvant chemotherapy (26% versus 28%, resp.) [17]. Finally, Pal et al. reviewed 24 patients who received GC and 22 who received MVAC with similar rates of pathologic complete responders (25% GC versus 22.5% MVAC) [18].

In our study, the proportion of patients found to achieve pT0 with neoadjuvant chemotherapy were consistent with prior studies. Patients who received GC and MVAC had pT0 rates of 29% versus 22%, respectively. Our data also takes into account clinical factors such as medical cardiac disease, baseline renal function, and the presence of complete resection and its possible effects on response rates. These clinical factors were not analyzed in any of the prior studies. We also examined overall pathologic response rates which were (P stage ≤ pT1) 56% and 45%, respectively, and found similar response rates between GC and MVAC. This is similar to the other studies reviewed (range ≤ pT1 36–58% GC and 35–50% MVAC) [15–18]. Based on prior studies suggesting that partial response to neoadjuvant chemotherapy improves survival, this data suggests that the majority of patients will derive benefit from receiving either GC or MVAC neoadjuvant chemotherapy [9, 10].

Our study has several limitations. First, it is a retrospective single institution review study and is therefore limited by the factors intrinsic to a retrospective study and as a result may limit the generalizability of our results. As this is not a randomized study, unmeasured confounders related to patient and provider factors associated with whether a patient received GC or MVAC cannot be excluded. However, having taken into account patient comorbidities in addition to clinical staging, our patients may represent a more homogenous population than other articles that did not delve into patient comorbidities. Our paper may represent a more accurate comparison between GC and MVAC chemotherapy. Patients that received GC were older than those that received MVAC, a finding that reflects the higher rates of toxicity associated with MVAC therapy and the trend to treat older patients with less toxic regimens. Limiting the analysis to those <70 years of age did not significantly change the results (data not shown). Another limitation is the small sample size of our cohort which may affect overall statistical significance of results, although our numbers are similar in size to those from other studies. Further, we have limited data on the relative dose intensity that each patient received and complications during chemotherapy, as many patients received chemotherapy outside of our institution. Finally, pathologic response rates, although associated with disease recurrence risk, are surrogate markers for outcome. Future analyses should explore disease-specific outcomes as a more robust endpoint.

Despite these limitations, our data adds to the growing literature showing similar response rates between neoadjuvant GC and MVAC [15–18]. It is our hope that studies such as these support the increased use of neoadjuvant chemotherapy to improve patient outcomes.

## Figures and Tables

**Table 1:** Patient demographics and clinical characteristics.

	Neoadjuvant chemotherapy		*P* value
	No *N* (%)	Yes *N* (%)	
Gender			0.87
Male	70 (76%)	66 (76%)	
Female	21 (24%)	21 (24%)	
Age			0.02
<60	21 (23%)	32 (37%)	
60–69	33 (36%)	35 (40%)	
>70	37 (41%)	20 (23%)	
Race			0.99
Caucasian	79 (89%)	75 (88%)	
Other	10 (11%)	10 (12%)	
Complete TURBT			0.28
Yes	32 (35%)	39 (46%)	
No	53 (58%)	43 (51%)	
Unknown	6 (7%)	3 (4%)	
Cardiac disease			0.13
Yes	23 (25%)	14 (16%)	
No	68 (75%)	73 (84%)	
Creatinine			0.49
≤1.0	47 (53%)	51 (59%)	
>1.0	41 (47%)	36 (41%)	

**Table 2:** Pathologic partial and complete response rates: neoadjuvant chemotherapy.

	Neoadjuvant chemotherapy		Odds ratio (95% CI)*
	Yes	No	
Complete response (pT0)			3.11 (1.45–6.64) *P* = 0.03
Yes	24 (28%)	8 (9%)	
No	63 (72%)	83 (91%)	
Any response (P ≤ T1)			3.23 (1.21–8.64) *P* = 0.01
Yes	45 (52%)	23 (25%)	
No	42 (48%)	68 (75%)	

*Adjusted for age, gender, race, clinical stage, prior BCG use, extent of TUR, cardiac disease, and baseline creatinine.

**Table 3:** Patient demographics and clinical characteristics for those who received GC or MVAC neoadjuvant chemotherapy.

	GC *N* (%)	MVAC *N* (%)	*P* value
Gender			0.10
Male	31 (76%)	28 (90%)	
Female	10 (24%)	3 (10%)	
Age			0.01
<60	13 (31%)	14 (45%)	
60–69	14 (34%)	16 (51%)	
>70	14 (34%)	1 (3%)	
Race			0.47
Caucasian	35 (85%)	29 (94%)	
Other	6 (15%)	2 (6%)	
Complete TURBT			0.72
Yes	16 (41%)	14 (45%)	
No	22 (59%)	15 (55%)	
Cardiac disease			0.25
Yes	8 (20%)	3 (10%)	
No	33 (80%)	28 (90%)	
Creatinine			0.24
≤1.0	28 (68%)	17 (53%)	
>1.0	13 (32%)	14 (47%)	
Clinical T-stage			0.52
T2	23 (56%)	14 (45%)	
T3	11 (27%)	9 (29%)	
T4	7 (17%)	8 (26%)	
Pathologic T-stage			0.45
pT0	12 (29%)	7 (23%)	
pTa	0 (0%)	2 (6%)	
pTIS	8 (20%)	2 (6%)	
pT1	3 (7%)	3 (10%)	
pT2	4 (10%)	6 (20%)	
pT3a	8 (20%)	6 (20%)	
pT3b	2 (5%)	2 (7%)	
pT4	4 (10%)	2 (7%)	
Pathologic N-stage			0.09
N0	35 (85%)	22 (71%)	
N1	6 (15%)	9 (28%)	

**Table 4:** Pathologic partial and complete response rates GC versus MVAC.

	Complete response rate (pT0)			
Neoadjuvant chemotherapy	Yes	No	*P* value	OR (95% CI)*
GC	12 (29%)	29 (71%)	0.52	1.00 (referent)
MVAC	7 (22%)	24 (78%)		0.39 (0.10–1.58)

	Complete + partial response rate (<pT1)			
Neoadjuvant chemotherapy	Yes	No		OR (95% CI)*

GC	20 (49%)	21 (51%)	0.26	1.00 (referent)
MVAC	11 (35%)	20 (62%)		0.45 (0.12–1.71)

*Adjusted for age, gender, race, clinical stage, completion of diagnostic resection, cardiac disease, and creatinine.
